# Eating Hubs in Multiple Sclerosis: Exploring the Relationship Between Mediterranean Diet and Disability Status in Italy

**DOI:** 10.3389/fnut.2022.882426

**Published:** 2022-06-16

**Authors:** Federica Felicetti, Silvia Tommasin, Maria Petracca, Laura De Giglio, Flavia Gurreri, Antonio Ianniello, Riccardo Nistri, Carlo Pozzilli, Serena Ruggieri

**Affiliations:** ^1^MS Center, Sant'Andrea Hospital, Rome, Italy; ^2^Department of Human Neurosciences, Sapienza University of Rome, Rome, Italy; ^3^Neuroimmunology Unit, IRCSS Fondazione Santa Lucia, Rome, Italy

**Keywords:** multiple sclerosis, Mediterranean Diet, lifestyle, disability, food network analysis

## Abstract

**Background:**

Multiple Sclerosis (MS) is a complex disease in which multiple factors contribute to disability accrual. Mediterranean Diet (MeDi) has shown beneficial effects across neurodegenerative diseases. We hypothesize that specific food habits, rather than global adherence to MeDi, might impact on MS. We aimed to (i) evaluate differences in adherence to MeDi between people living with MS (PwMS) and healthy controls (HC); (ii) characterize eating patterns in PwMS and HC, identifying the most influential MeDi items for each group by the use of network analysis; (iii) explore the relationship between patients' eating habits and disability.

**Materials and Methods:**

In this cross-sectional study, we consecutively recruited 424 PwMS and 165 matched HC. Data were obtained through the administration of self-reported questionnaires. Expanded Disability Status Scale (EDSS) and Fatigue Severity Scale (FSS) were evaluated in the MS population. We performed between-groups comparisons via unpaired two-sample *t*-test and X^2^ test as appropriate. We calculated food networks in both MS cases and HC using and tested the association between hub nodes and disability. Finally, we conducted a *post-hoc* analysis, investigating the relationship between food items, lifestyle factors (smoking, exercise) and clinical outcomes.

**Results:**

Most participants adhered sufficiently to MeDi. Exploring each group separately, fruit, vegetables, cereal, and fish were identified as hubs in PwMS, while meat and alcohol were identified as hubs in HC. Hubs were all inter-correlated, indicating that eating habits of PwMS include a large intake of all the foods identified as hubs. EDSS was predicted by the intake of vegetables (beta = −0.36, *p* < 0.03) and fish (beta = −0.34, *p* < 0.02). The model including smoking pack/year, International Physical Activity Questionnaire (IPAQ) score and intake of “negative foods” predicted 6% of the variance in EDSS (*p* < 0.001), while the model including smoking pack/year and IPAQ score predicted 4% of the variance in FSS (*p* < 0.001).

**Conclusions:**

We identified a sufficient adherence to MeDi in our population. PwMS showed overall a healthier dietary pattern than HC. Vegetables and fish intake were associated with disability outcomes. Future longitudinal studies applying integrated approaches are needed to understand lifestyle added value to the use of standard pharmacological therapies.

## Introduction

Multiple Sclerosis (MS) is a complex disorder with genetic, immunological and environmental factors contributing to the disease onset and evolution. Among environmental factors, viral infections, vitamin D deficiency, childhood obesity, smoking, incorrect dietary habits, and vascular risk factors might play a role in the development of MS (1). Whether dietary habits and lifestyle affect the course of MS is still a matter of discussion and established MS therapies are not usually integrated by specific indications on diet. Nonetheless, the known effects on the disease evolution of migration, low vitamin D levels and obesity during adolescence make a strong case for the relevance of dietary habits in MS (2). A recent study based on web interviews provided data on dietary behavior of more than 7,000 MS patients, demonstrating a strong association between healthy dietary habits, better physical and mental outcomes and lower level of disability (3). On the other hand, unhealthy lifestyle habits and their consequences, such as being overweight or obese, smoking and sedentary life, are common in MS population with unfavorable sequelae (4). Among healthy dietary regimens, the traditional Mediterranean Diet (MeDi), has shown beneficial effects across neurodegenerative diseases (5). Dietary supplementation and education on food intake, with a particular focus on MeDi components, has shown a positive impact on quality of life and cognitive performance in patients with Alzheimer's disease (6), on motor symptoms in Parkinson's disease (7) and on global disability in amyotrophic lateral sclerosis (8). Indeed, a modified MeDi approach has recently been associated to improvements in fatigue and other MS symptoms, as well as disability (9). MeDi has shown promising results not only on health-related issues but also on cognitive performance sustained by brain volume preservation in MS. Particularly in a cross-sectional study of patients with early MS, higher hybrid MeDi score correlated with preserved thalamic volumes (10) a site known to be crucial for cognitive decline (11). Such beneficial effects are likely mediated by the antioxidant components of MeDi, counteracting the oxidative stress secondary to mitochondrial dysfunction, which represent a pathological hallmark in all neurodegenerative disorders (12, 13). MeDi is characterized by consumption of fruit, vegetables, whole grains, legumes, nuts, lean fish, dairy products, white meat, small quantities of red meat, moderate consumption of alcohol and extra virgin olive oil as a fat source. These foods not only exert an antioxidant action due to their composition but can positively influence the function of immune cells in MS, probably favoring a shift toward an anti-inflammatory profile (14).

Given these premises, MeDi seems a good candidate for a dietary intervention in People Living with MS (PwMS). However, before suggesting specific dietary indications, a deeper knowledge of the dietary patterns commonly followed by PwMS, should be acquired. This is not trivial as, even though Italy belongs to the Mediterranean basin, a region where high level of adherence to MeDi is expected, surveys on eating behavior of adult population revealed that only one third of the population had an adequate intake of vegetables and fish, and the energy intake from saturated fats and sugars was globally very high (15).

Additionally, even in populations showing an overall good adherence to MeDi, relative consumption of specific foods, as well as the relationship between different food categories within the diet (i.e., consumption of one food driven or associated to the consumption of other foods) might differ across subgroups.

We hypothesize that the consumption of specific food items, or their eating pattern, rather than the global adherence to MeDi (16), might characterize MS patients and potentially affect their disability outcomes. Indeed, when studying diet behaviors, it is crucial to evaluate the multifaceted interdependence of foods in the habitual diet. In this context a recent work has implemented network science tools to identify novel diet patterns in prodromal dementia (17), showing how network methods may progress our knowledge of associated risk factors for complex disease as dementia and MS. With network analysis we can recognize eating hubs (i.e., items that are consumed in association with many other items within the dietary regimen), thus highlighting complex relations, hidden in the eating behavior, that may be related to patients' clinical status. This analysis would be complementary to the wide investigations on dietary habits, that have been performed and are still ongoing, and that so far have elucidated the relation between incorrect dietary behavior and MS poor clinical status and progression (3).

Given these premises, we set out to explore dietary patterns and their relationship with disability outcomes in a sample of PwMS followed in a large tertiary center in Central Italy, implementing a novel network analysis approach. Additionally, as diet is only one of the lifestyle habits potentially affecting disability outcomes in MS, our analysis was complemented by the exploration of aspects such as smoking and physical exercise (9).

Specifically, the aims of this work were (i) to evaluate differences in adherence to MeDi, intake of specific food, lifestyle habits between PwMS and HC; (ii) to describe the dietary pattern of PwMS and HC identifying the most influential demographic or MeDi items that characterize each group's eating habits (i.e., eating hubs); (iii) to explore the relationships between those hubs and disability outcomes.

## Methods

### Participants

Participants older than 18 at the time of screening were prospectively enrolled at the MS Center of the Sant'Andrea Hospital in Rome between March 2018 and August 2019. Participants with a diagnosis of MS according to most recent revised McDonald criteria 2017 (18) regardless of disease phenotype were recruited together with their family members and hospital staff to act as control group. The ethical committee board of Sapienza University of Rome at Sant'Andrea Hospital provided approval for the project. Informed, written consent has been obtained in all participants.

Exclusion criteria for both groups were: (1) diagnosis of metabolic diseases; (2) mental or psychiatric diseases; (3) pregnancy or breastfeeding; (4) food allergies and food intolerances; (5) ongoing vegetarian or vegan diet; (6) any condition preventing participants to provide adequate answers to the administered questionnaires; (7) ability to walk without support/aid. For latter exclusion criteria, PwMS with and Expanded Disability Status Scale (EDSS) (19) higher than 5.5 were not recruited.

### Study Procedures

The following information were collected in PwMS and HC: demographic data, pharmacological therapies, smoking (number of cigarettes smoked per day and duration of habit, i.e., smoking pack/year), telephone and email contacts.

We also evaluated the following anthropometric indicators: weight and height to calculate Body Mass Index (BMI kg/m^2^), waist (WC) and hip circumferences, waist-hip ratio (WHR).

For BMI, we used World Health Organization (WHO) cut-points to asses underweight (<18.5 kg/m^2^), normal weight (18.5–24.9 kg/m^2^), overweight (25.0–29.9 kg/m^2^), and obese (>30 kg/m^2^). For men WC≥94 cm and for women WC≥80 cm are cut-points used to assess cardiovascular risk in the general population (20). Fat distribution is further defined as WHR above 0.90 for males and above 0.85 for females (21).

Subsequently, the following questionnaires were administered:

- MeDi adequacy questionnaire (22). This is a nine-question test, one for each food group (fruit, vegetables, legumes, cereals, fish, meat and cold cuts, milk and derivatives, olive oil, alcohol). For each question there are three different answers, regarding the frequency intake of those particular foods, daily or weekly, with a score from 0 to 2, begin 0 = no intake and 2 = more than once a week, referring to the last month. Adding all the results, we evaluated the adequacy to MeDi (0–4 not adequate; 5–9 poorly adequate; 10–15 sufficiently adequate; 16–18 completely adequate). Moreover, we computed the frequency of intake for each food item and grouped food categories as “positive” (fruit, vegetables, legumes, cereals, fish, olive oil) vs. “negative” (meat and cold cuts, milk and derivatives, alcohol): the higher the score in the “positive” group the better the diet of the participant, while the higher score in the “negative” group the worse the nutrition habits.
- The reduced form of the International Physical Activity Questionnaire (IPAQ) assessing the engagement in physical activity (23). The questionnaire is divided into 4 sections in which intense activities, moderate activities, walking and sitting are distinguished. For each of these categories the participant needs to specify how many days per week the various activities had been carried out and the total minutes of activity in one of those 7 days referring to the previous week. According to the scores obtained in the test, the person falls into three different groups that specify the levels of physical activity: inactive (lower score 700 Met); sufficiently active (score between 700 and 2,519 Met) and active or very active (score higher than 2,520 Met).

For each PwMS, the respective level of disability as measured by EDSS (and fatigue in terms of Fatigue Severity Scale (FSS) (24) were evaluated the same day of questionnaire completion.

PwMS were dichotomized into two subgroups according to their EDSS score: no disability (EDSS ≤1.5; i.e., no or minimal signs in more than one functional system) and disability (EDSS ≥2).

### Network Analysis

To identify what demographic or MeDi items were most influential for the eating habits of PwMS and how they were linked to disability and fatigue, we performed a network analysis. We created a structure depicting the connections, namely edges, among all the variables of interest, namely nodes, such as age, sex, and MeDi items (fruit, vegetables, legumes, cereals, fish, meat, dairy, olive oil, alcohol). Thanks to this representation, we were able to identify the hubs, that are the most connected variables, thus the variables with the highest amount of connections with the other variables. First, to generate the edges connecting the nodes of the network, we built a mutual information matrix, representing the relation among age, sex, and MeDi items for both patients and HC (17). The mutual information matrix was selected to describe the relation among age, sex and MeDi items, as it provided information on the mutual dependence between couples of variables, without any a priori assumption of linear association among them. Since the investigated variables were all categorical except for age, the continuous variable age was grouped by ranges of years and devised in 5 classes (from class 0 if age <20 years to class 4 if age>49 years and the other classes covering 10 years each) to build the mutual information matrix.

Then, we investigated the difference of age, sex and MeDi items association between PwMS and HC We obtained 1,000 random matrices by reshuffling, 1,000 times, values taken from the information matrices of PwMS and HC, and placing them into two matrices, that had the same dimensionality of the mutual information matrices but casual entries, and that were subtracted one from the other. Then, we averaged these 1,000 random matrices and compared the result with the difference between the mutual information matrix of HC and that of PwMS, by means of a Z-score. Z-scores were considered significant at alpha = 0.05 if Z>|1.96|.

**Figure 1:**
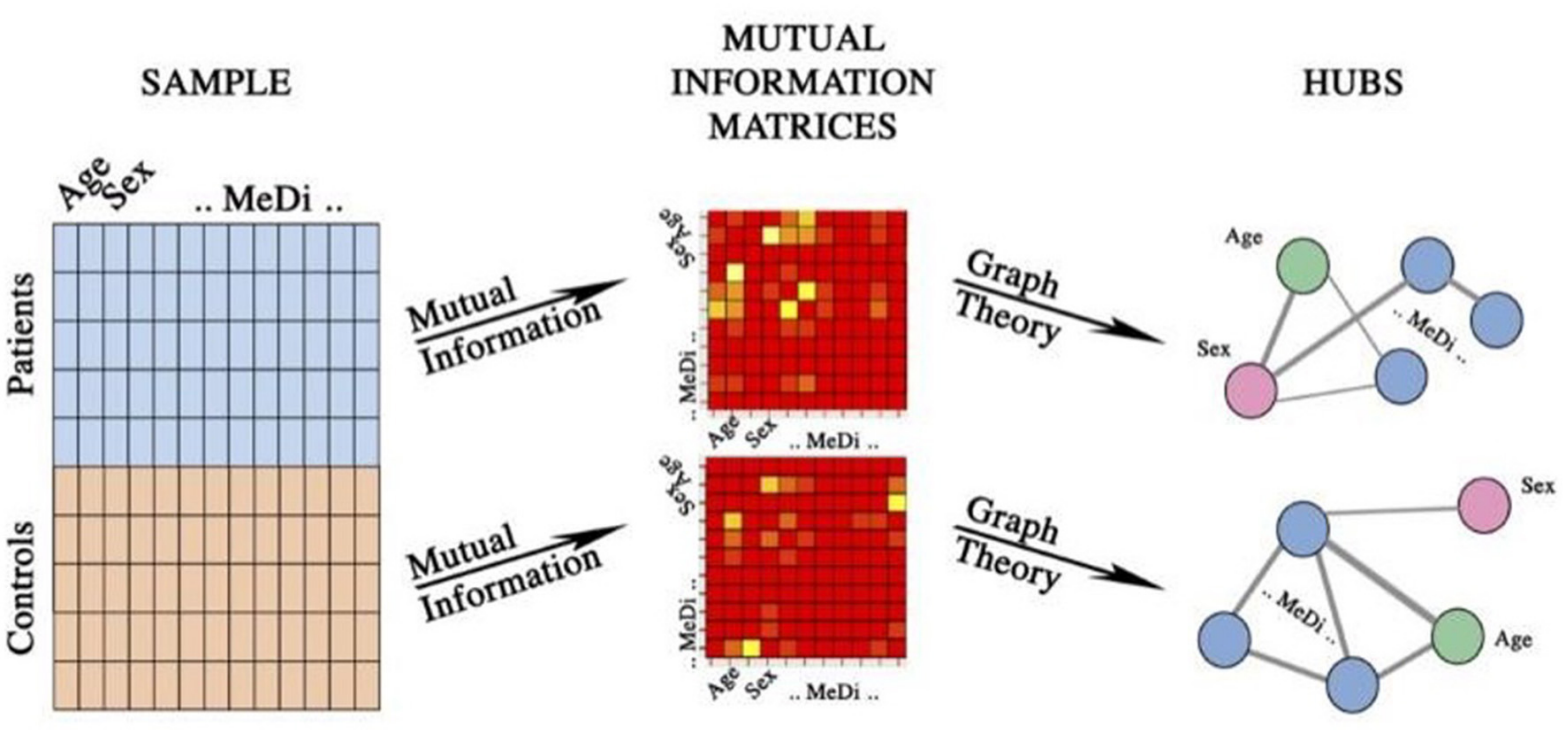
Flowchart of the network analysis. Age, sex, and Mediterranean Diet items, such as fruit, vegetables, legumes, cereals, fish, meat, diary, olive oil, and alcohol, were collected for both people living with MS and healthy controls. From the two datasets the mutual information matrices were calculated and hubs were identified via graph theory.

Moreover, on the mutual information matrices of both PwMS and HCs, we calculated the hubs. Analytically, in a network, hubs are the variables with the degree larger than the average plus a standard deviation. The degree of an item is the number of its neighbors, e.g., of the number of links incident upon it. Network analysis was implemented in the open-source R environment (https://www.r-project.org/. For a flowchart of the network analysis see Figure 1.

### Statistical Analysis

All values are presented as mean (standard deviation) or median [range], as appropriate. Normal distribution assumption was checked for continuous variables by using Shapiro-Wilk tests.

Unpaired two-sample *t*-test and X^2^ test were used to compare demographic, clinical, anthropometric indicators, adherence to MeDi, frequency of food intake and IPAQ scores between PwMS MS and HC, as well as between PwMS with different levels of disability, as appropriate.

To test the association between hub nodes from network analysis and disability, a multivariate logistic regression was conducted. Specifically, we included hubs (obtained by network analysis) as independent variable and scores of either disability or fatigue as dependent variable. Classes of disability were devised as no disability (class 0, EDSS < = 1.5), or disability (class 1, EDSS>1.5). Similarly, classes of fatigue were devised as no fatigue (class 0, FSS lower than the median value) or fatigue (class 1, FSS higher than the median value). Initially, we performed a sensitivity analysis to evaluate the minimum regression coefficient (beta) relative to sample size, considering alpha = 0.05 and power = 0.90. In multiple regression, collinearity exists if one variable is linearly predicted from the other and it may affect the reliability of the calculation of regression coefficients. Therefore, we checked for collinearity via Spearman correlation and removed correlated variables (*p* < 0.05 not corrected for multiple comparisons to be conservative at the most). However, for completeness, also association between dependent variables and removed hubs was calculated as bivariate logistic regression. Significance was reached if *p* < 0.05.

Finally, as the network analysis identified as hubs only items pertaining to the “positive foods,” and did not include lifestyle factors other than diet, we conducted a *post-hoc* analysis, investigating the relationship between “negative foods,” lifestyle factors (smoking, exercise) and clinical outcomes (EDSS and FSS) via hierarchical regression.

Analyses were conducted with the Statistical Package for Social Sciences 25.0 (SPSS, Chicago, IL, USA). Network statistical analyses were implemented in the open-source R environment (https://www.r-project.org/. Sensitivity analysis was performed with G-power (https://stats.idre.ucla.edu/other/gpower/.)

## Results

### Participants in the Study

**Figure 2:**
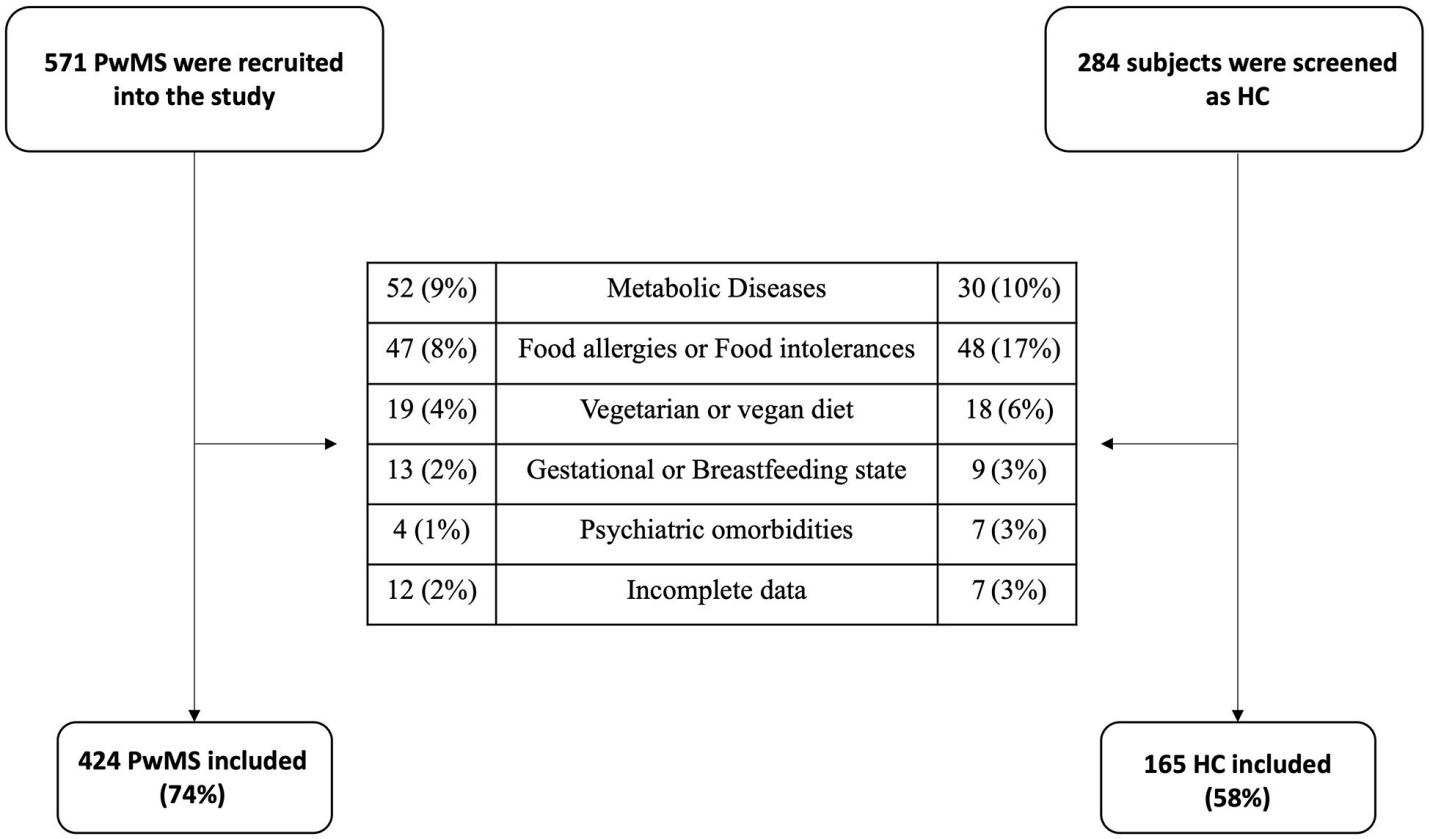
Flowchart of the participants in the study. PwMS, People Living with MS; HC, Healthy Controls.

From an initial screening of 571 PwMS we enrolled 424 (74%) participants. Among the excluded patients there were 52 PwMS with metabolic diseases (9%), 47 with food allergies or food intolerances (8%), 19 people following a vegetarian or vegan diet (4%), 13 women in a gestational or breastfeeding state (2%), 12 (2%) have not been included due to incomplete data and 4 (1%) patients had psychiatric comorbidities. Two hundred and eighty four participants were screened as HC, of which 165 people (58%) were found to be suitable for study inclusion. Among the excluded participants there were 30 people with metabolic diseases (10%); 48 with food allergies or intolerances (17%); 18 people following a vegetarian or vegan diet (6%), 9 women in a gestational or breastfeeding state (3%), 7 have not been included due to incomplete data (3%) and 7 patients had psychiatric comorbidities (3%). For a flowchart of the study participants please see Figure 2.

**Figure 3:**
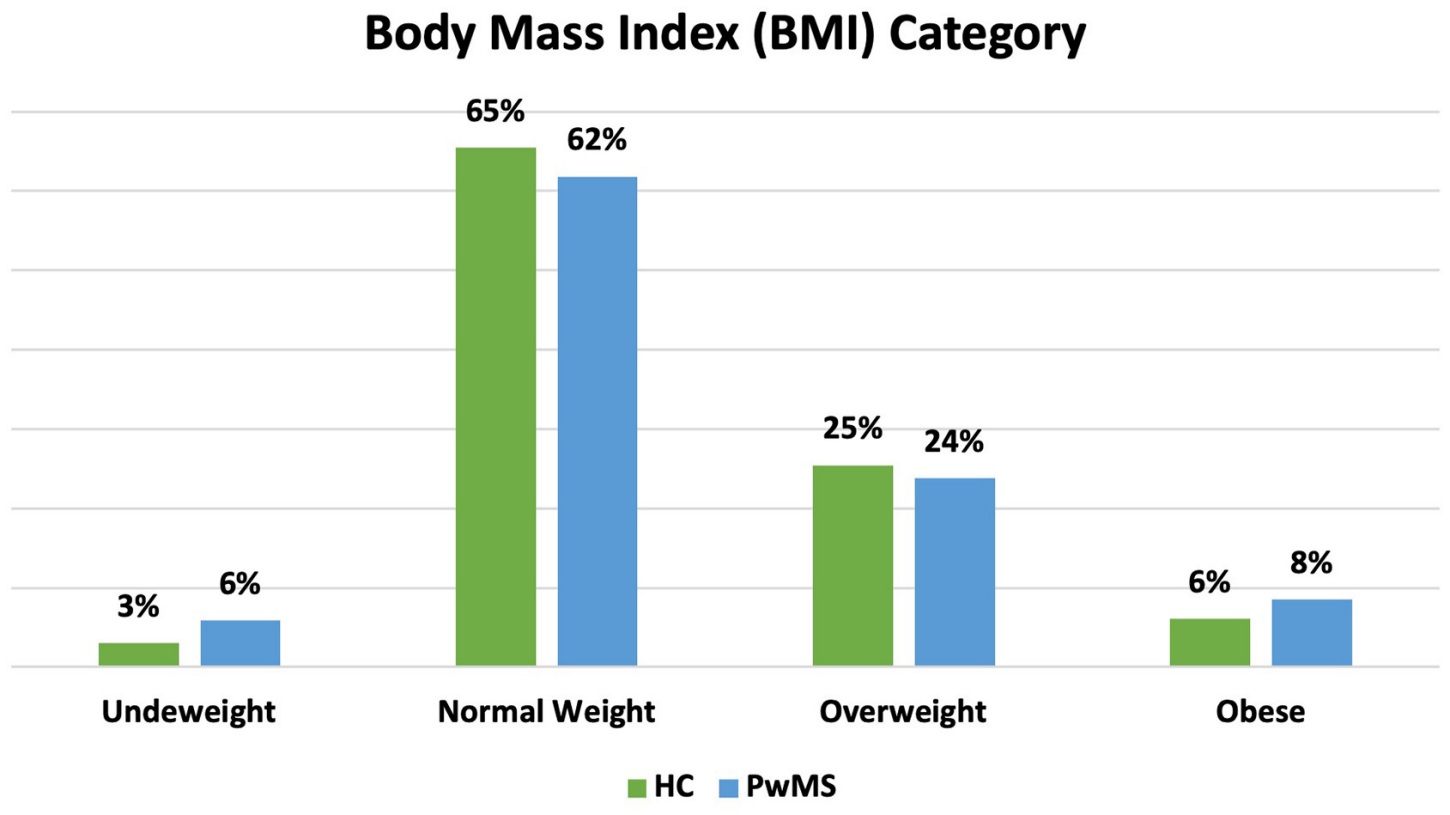
Percentage of participants according to Body Mass Index (BMI) Category. Underweight (<18.5 kg/m^2^); normal weight (18.5–24.9 kg/m^2^); Overweight (25.0–29.9 kg/m^2^) and obese (>30 kg/m^2^). PwMS, People Living with MS; HC, Healthy Controls.

Table 1 summarizes the main demographic characteristics and anthropometric measurements of the two examined groups. PwMS showed higher values of hip circumference compared to HC (*p* < 0.014), while there was a trend for waist circumference (*p* < 0.069) and no differences in terms of WHR and BMI. Among study participants only men in the HC group had BMI values belonging to the overweight range. No differences in terms of frequencies among the two groups were detected (Figure 3). Male HC subjects had WHR values above the cut-off, while all the other participants were normal weighted with normal WHR values. All subjects in our study were within the WC cut-off values.

**Table 1:** Demographical and anthropometric variables of the study participants.

	**People living with MS (*N =* 424)**	**Healthy Controls (*N =* 165)**	***P*** **-values**
Female/Male (%)	284 (67)/140 (33)	112 (68)/53 (32)	0.8
Age, years [range]	42.6 (11.0) [18-74]	40.9 (14.5) [18-77]	0.127
BMI, kg/m^2^	23.8 (3.9)	23.7 (3.5)	0.68
Female	23.3 (4.3)	22.9 (3.4)	0.33
Male	24.8 (3.0)	25.3 (3.1)	0.29
Waist circumference, cm	83.2 (12.3)	81.0 (14.8)	0.069
Female	80.0 (12.3)	76.8 (13.2)	**0.024**
Male	89.6 (9.5)	89.8 (14.2)	0.90
Hip circumference, cm	100.7 (8.8)	98.4 (13.2)	**0.014**
Female	100.4 (9.9)	98.7 (13.4)	0.18
Male	101.3 (5.6)	97.7 (12.6)	**<0.01**
Waist-hip ratio (WHR)	0.82 (0.08)	0.82 (0.1)	0.8
Female	0.79 (0.07)	0.78 (0.09)	0.10
Male	0.88 (0.07)	0.91 (0.08)	**0.022**

*Values are expressed as mean. Standard Deviation (SD) is indicated in the round brackets. The minimum and maximum values of the measurements are indicated in the square brackets [range]. P-values refer to comparisons between groups; significance was reached if p < 0.05 and expressed in bold*.

Table 2 shows the main clinical characteristics and anthropometric measurements of the PwMS subgroups. PwMS in the disability group were older, had longer disease duration as well as higher FSS scores compared to PwMS in the no disability group. Waist circumferences and WHR were significantly different among the two subgroups.

**Table 2:** Clinical and anthropometric characteristics of people living with MS involved in the study, according to level of disability.

	**Total (*N =* 424)**	**EDSS ≤1.5 (*N =* 205)**	**EDSS ≥2 (*N =* 219)**	***P***
**Clinical characteristics**				
Age, years	42.6 (11.0)	39.3 (10.5)	45.6 (10.7)	<0.001
Age of onset, years	31.4 (9.9)	30.1(8.7)	32.7 (10.8)	<0.01
Disease duration, years	12.0 (8.4)	10.1 (7.3)	13.8 (9.0)	<0.001
Therapies (no/first line/second line), %	20/60/20	20/64/16	20/54/26/	n.s.
Disease course, Relapsing Remitting (RR)/Secondary Progressive (SP)	402/22	205/0	198/22	<0.001
Median Expanded Disability Status Scale (EDSS) [range]	2.0 [0–5.5]	1.5 [0–1.5]	3 [2–5.5]	<0.001
Fatigue Severity Scale (FSS)	3.8 (1.7)	3.1 (1.6)	4.5 (1.5)	<0.001
**Anthropometric characteristics**				
BMI, kg/m^2^	23.8 (3.9)	23.65 (4.05)	24.07 (3.94)	0.270
Waist circumference, cm	83.2 (12.3)	81.8 (11.9)	84.6 (12.6)	**0.021**
Hip circumference, cm	100.7 (8.8)	100.3 (8.55)	101.2 (9, 3)	0.281
Waist-hip ratio (WHR)	0.82 (0.08)	0.81 (0.78)	0.83 (0.08)	**0.013**

*Values are expressed as mean, unless otherwise specified. Standard Deviation (SD) is indicated in the round brackets. The minimum and maximum values of the measurements are indicated in the square brackets. P-values refer to comparisons between groups; significance was reached if p < 0.05 and expressed in bold*.

*First line therapies: interferon, glatiramer acetate, dimethyl fumarate, teriflunomide*.

*Second Line therapies: fingolimod, natalizumab, ocrelizumab, alemtuzumab*.

### Adherence to MeDi, Intake of Specific Food, Lifestyle in PwMS and HC

The majority of participants in each group adhered sufficiently to MeDi (60% in the group of PwMS and 56% in HC group). In the PwMS group, only one participant had an inadequate diet, while 38% poorly adequate and 2% completely adequate diet. Among HC, 2% had inadequate adherence to MeDi, 41% of participants showed poor adherence, while 1% followed a completely adequate diet. There were no significant differences in terms of adequacy to MeDi between PwMS and HC. Participants less adherent to MeDi showed higher smoking pack/years values in the whole sample (*p* < 0.001) as well in the PwMS (*p* < 0.001).

**Figure 4:**
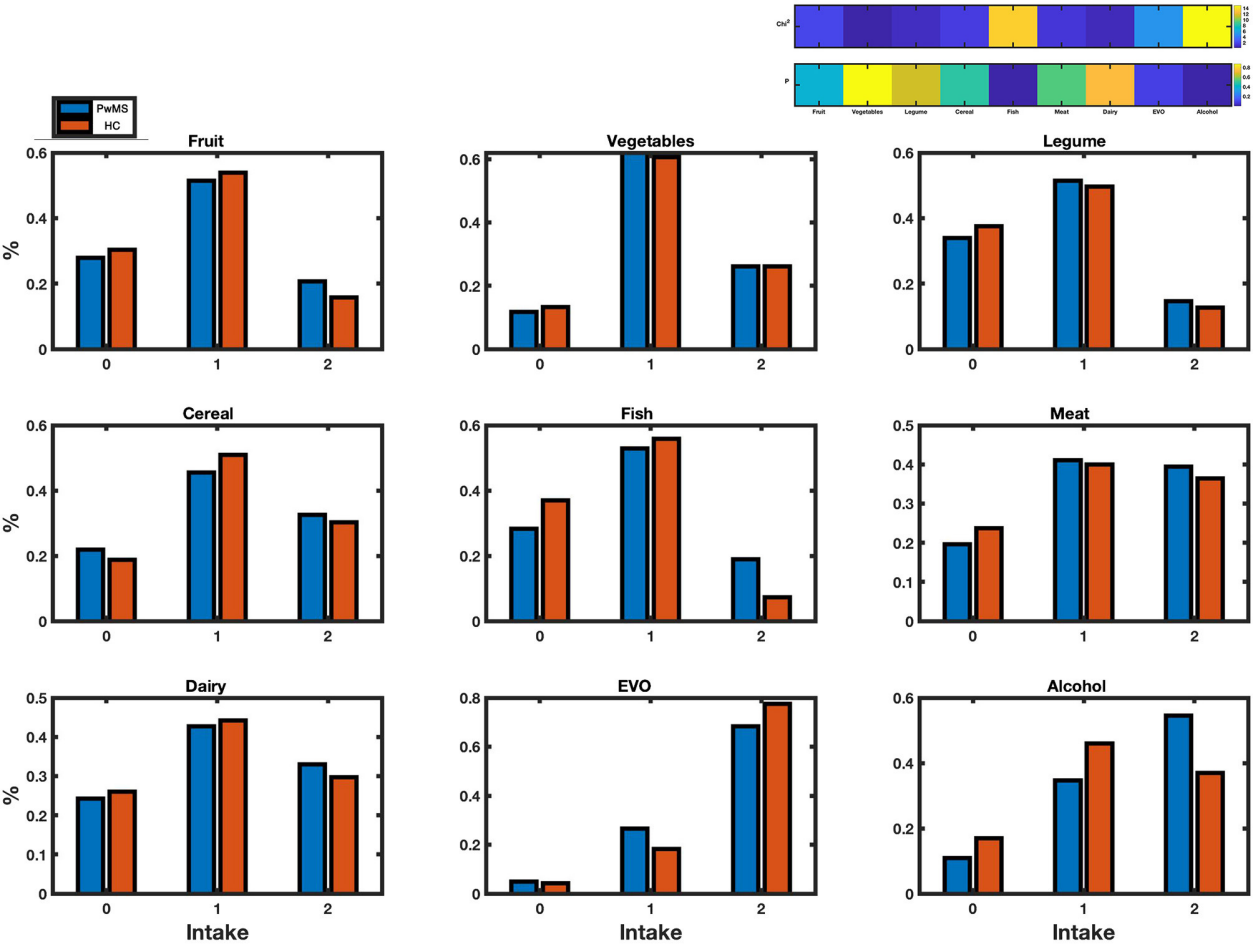
Food intake frequency. Histograms of food intake frequency of people living with multiple sclerosis (PwMS, blue) and healthy controls (HC, orange), devised by intake scores (0, 1, 2). On the top right, heatmaps display X^2^ and relative *p*-value of food intake difference between PwMS and HC. Significant difference (*p*-value < 0.05) was found for fish and alcohol alone. Chi^2^: X^2^; EVO: olive oil.

When we explored differences in terms of food intake frequency, we observed a tendency to a healthier diet in the group of PwMS, as they consumed more frequently fish and less frequently alcohol than HC (*p* < 0.001) (Figure 4).

**Figure 5:**
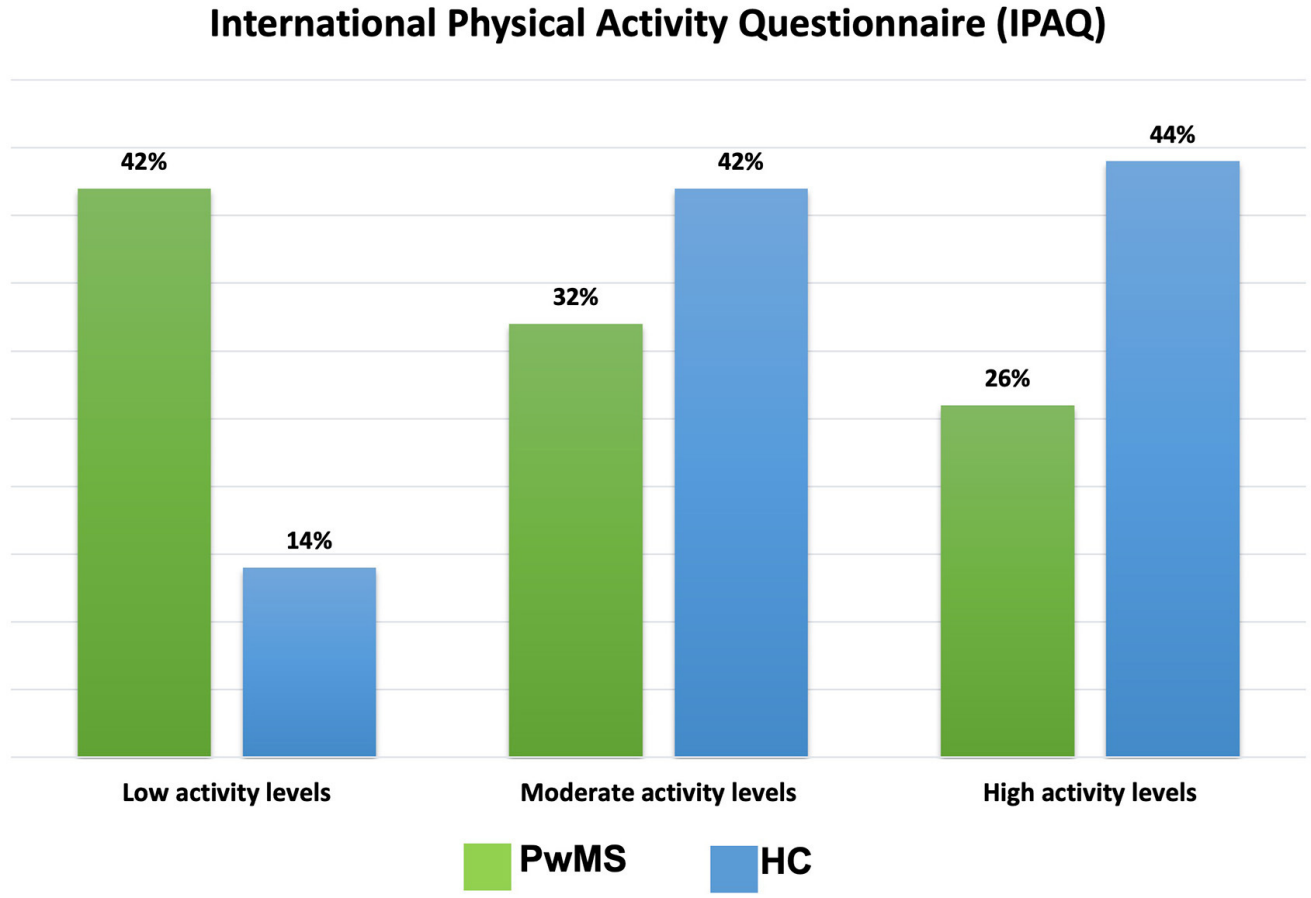
Percentage of participants according to group for each category of International Physical Activity Questionnaire (IPAQ). PwMS, People Living with MS; HC, Healthy Controls.

PwMS showed overall lower IPAQ scores (2227.21 ± 3587.26) compared to HC (3701.33 ± 8338.35) [*p* = 0.03]. Figure 5 shows the percentage of participants for each category according to groups.

In the group of PwMS there was a greater proportion of participants with a long-time smoking habit compared to HC (4.2 ± 8.6 vs. 1.0 ± 3.1, *p* < 0.01).

There were no differences in terms of adherence to MeDi between the two PwMS subgroups. However, when we compared the frequency of food intake, a greater proportion of participants in the no disability subgroup consumed fish more frequently than patients in the disability subgroup (*p* < 0.01).

PwMS in the no disability subgroup engaged in physical activities more often and more intensely compared to those with disability (2667.8 ± 4263.6 vs. 1814.7 ± 2757.7, *p* = 0.014).

PwMS in the no disability group had lower values of smoking pack/years compared to those with disability (2.9 ± 6.4 vs. 5.5 ± 10.1, *p* < 0.002).

### Food Network Analysis

**Figure 6:**
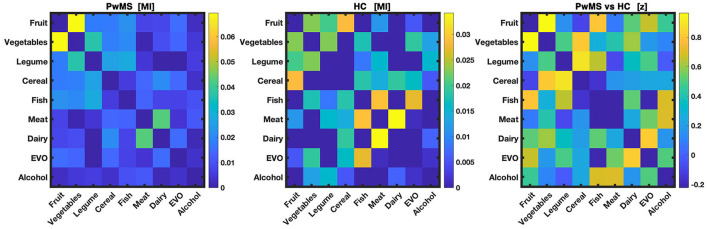
Mutual information matrices. Mutual information matrices of people living with MS (PwMS) and healthy controls (HC), as well the matrix resulting by the 2-sample analysis to investigate differences between PwMS vs. HC and reporting the z-score, are displayed from the left to the right. PwMS vs. HC have no intake connectivity difference in any of the food-pair (z-score > 1.96).

**Figure 7:**
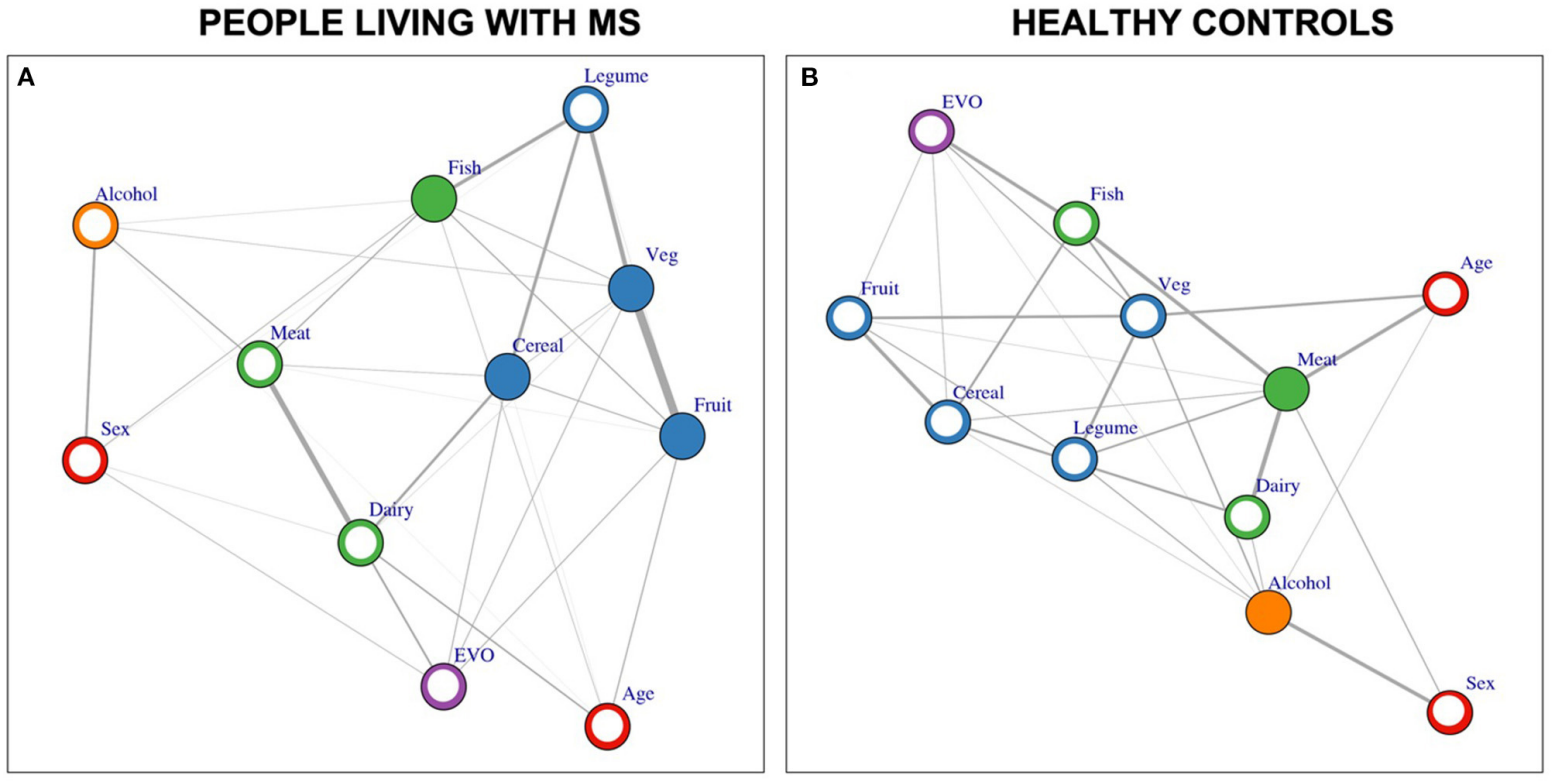
Demographic and food networks. Demographic data, such as age and sex, and Mediterranean Diet food items, such as cereal, vegetables, fruit, diary, meat, fish, olive oil (EVO) are displayed as nodes of a graph, whose links (edges) are calculated as mutual information coefficients for either people living with MS **(A)** and healthy controls **(B)**. Hubs are the variables with the highest amount of connections with the other data and represent the mos influential items for the eating habits of people living with MS and controls. Link thickness mirrors the intensity of the mutual information coefficient between the linked nodes. Circles display nodes and are colored following the class, thus red if demographics, blue if with vegetal origin, green if with animal origin, purple for EVO, orange for alcohol. Full circles identify hubs.

We found no difference between mutual information matrices of PwMS and HC (Figure 6). When exploring each group separately, the network of PwMS identified fruit, vegetables, cereal and fish as hubs (Figure 7A), while HC's network identified meat and alcohol as hubs (Figure 7B).

Beta = |0.32| was the minimum beta evaluable with a sample of 424 PwMS. Hubs were all inter-correlated, as shown in Table 3. Significant positive Spearman correlations show monotonic association between variables, thus that the increase of one is paralleled by the increase of the other, thus eating habits of PwMS include a large intake of all the “positive foods” identified as hubs.

**Table 3:** Spearman correlation among MeDi item intake identified as hubs in people living with MS.

	**Fruit**	**Vegetables**	**Cereal**	**Fish**
Fruit	–	**0.33 (0.001)**	**0.13 (0.01)**	**0.22 (0.001)**
Vegetables		–	**0.19 (0.001)**	**0.21 (0.001)**
Cereal			–	**0.12 (0.01)**
Fish			0.12	–

*To check for collinearity among hubs, we calculated Spearman correlation between couples of items, as reported in the table. Significant correlations are reported in bold font, relative p-value is shown between round brackets*.

We found no difference between mutual information matrices of PwMS subgroups classified by EDSS or FSS.

Because of the collinearity, we did not perform a multiple regression including all the hubs in the same model, but rather a bivariate logistic regression for each hub to predict either EDSS or FSS groups. EDSS was predicted by the intake of vegetables (beta = −0.36, *p* < 0.03) and fish (beta = −0.34, *p* < 0.02). FSS was not predicted by any hub, e.g., fruit, vegetables, cereal and fish.

### Relationship Between “Negative Foods” Lifestyle Factors and Disability

When testing the relationship between “negative foods” diet and disability, the model including smoking pack/year, IPAQ score and intake of “negative foods” predicted 6% of the variance in EDSS (*p* < 0.001), with smoking pack/year, exercise and diet being independent contributors (beta = 0.190, *p* < 0.001; beta = −0.138, *p* < 0.004; beta = 0.098, *p* < 0.039, respectively).

The model including smoking pack/year and IPAQ score predicted 4% of the variance in FSS (*p* < 0.001), with smoking pack/year and exercise being independent contributors (beta = 0.135, *p* < 0.005; beta = −0.169, *p* < 0.001, respectively).

## Discussion

In our work we did not find significant differences in terms of adequacy to MeDi between PwMS and HC, nor significant differences regarding food intake within the study population besides fish and alcohol consumption. Even though most participants fell into the normal weight category value according to BMI and were adherent to MeDi, PwMS showed higher values of waits and hip circumferences compared to HC. The network analysis allowed us to unveil the relation between specific food items (fruit, vegetables, cereal and fish). In particular, PwMS showed an eating behavior characterized by the consumption of “positive” foods, whose intake appears highly intercorrelated and might partially contribute to physical disability level.

### Adherence to MeDi, Intake of Specific Food, Lifestyle Habits

We found no significant differences in MeDi adequacy between PwMS and HC, in line with previous findings (25). However, although most of the participants in both groups were sufficiently adherent to MeDi, a very low percentage of participants were completely adherent to it (2% PwMS and 1% HC). Even though MeDi is the most used food model in Mediterranean countries, such as Italy, even the general population is not completely adherent to it, with younger age groups and smokers being less adherent (26). Accordingly, in our work participants less adherent to MeDi showed higher values of smoking pack/years and particularly, in the group of PwMS there was a higher percentage of participants with long-term smoking habits compared to HC. Generally, the category of smokers seems to have a less healthy lifestyle and incorrect eating habits than non-smokers (27).

When we explored differences in terms of food intake frequency, we observed a tendency toward a healthier diet in the group of PwMS, as they consumed more frequently fish and less frequently alcohol than HC. Despite the healthier diet, in our sample of PwMS higher values of waist and hip circumferences compared to HC were detected. WC is used to measure abdominal adiposity and high values are common among PwMS (28). While in our study population the BMI was in the range of accepted values and most of the PwMS fell in the normal weight category, there is evidence showing that an increase in circumferences values is associated with greater disability even in normal weight subjects (28). This is confirmed in our sample, where patients with higher level of disability showed significantly higher WC and WHR values in comparison with the no disability group. An excess of visceral or abdominal adiposity is one of the characteristics of the metabolic syndrome often related to other disorders such as diabetes, hyperlipidemia and hypertension (28). Metabolic and vascular comorbidities affect both neuroperformance and brain and gray matter volumes in MS, contributing to neurodegeneration and long-term disability (29).

Even though in our work we did not collect serological nutrional biomarker and such interpretations remain speculative, several evidences support the correlation between an excess of adipose tissue and the severity of MS. Firstly, an overweight or obese subject has a state of chronic inflammation characterized by an altered production of cytokines, such as IL-6, TNF-α, leptin, and a downregulation of anti-inflammatory molecules (30). Additionally, overweight negatively impacts disease course by modulating monocyte cell number through ceramide-induced DNA methylation of anti-proliferative genes (31). This increased state of inflammation of overweight/obese subjects is also observed within the Central Nervous System (CNS). In particular, a recent study has shown elevated levels of proinflammatory molecules (IL-6 and leptin) and reduced levels of the anti-inflammatory cytokine IL-13 in the cerebrospinal fluid (CSF) of obese MS patients, and patients with higher BMI (BMI > 30 kg/m^2^) also had significantly higher EDSS values (30).

Excess of adipose tissue in PwMS might also affect daily clinical practice, as it can interfere with the response to drugs, possibly due to altered drug pharmacokinetics (32). Indeed, a study carried out on a population of adult patients, under interferon-beta (IFNβ) therapy, showed that overweight and obese patients have increased disease activity, as assessed by NEDA status (composite score of no evidence of disease activity). This result indicates that overweight and obesity may have an impact on IFNβ-treatment response (33). Based on these data, regular exercise by reducing visceral adiposity may play a key role in reducing inflammation. Finally, in line with previous evidence (34), PwMS were less physically active and showed overall lower IPAQ scores compared to HC.

### Food Network Analysis

The food network analysis identified fruits, vegetables, cereals and fish as hubs in PwMS with meat and alcohol being hubs in HC, suggesting, in line with our analysis of food intake frequency, that PwMS tend to have a healthier diet than HC. It is possible that PwMS, having a chronic disease, are urged to pay more attention to what they eat than the general population. Indeed, a previous study has shown that patients, following an initial clinical diagnosis of CNS demyelination, tend to change their diet, increasing the amount of fruit and/or vegetables and following a low-fat diet (35). Additionally, a recent work has shown a trend toward reduced levels of storage lipids (fatty acids, cholesterol esters, triglycerides, and diglycerides) in the plasma of PwMS in comparison with HC (36). Additionally, in line with previous findings highlighting the potential effects of MeDi on MS course and disability (16) in our study participants higher consumption of vegetables and fish was inversely correlated to global motor disability, as assessed via EDSS. This relationship hints to a beneficial role of food hubs on disability outcomes but might also disclose an inverse causality.

Foods identified as hub of PwMS (fruits, vegetables, cereals and fish) are rich in fiber, vitamins, ω-3 polyunsaturated fats or antioxidant molecules and possibly exert a protective effect on the disease (14, 37). In particular, antioxidant molecules such as vitamins C and E, plant polyphenol and carotenoids might contrast free radicals, with beneficial effects on the inflammatory response (37). Another mechanism by which hub foods that could influence the disease outcomes is the modulation of the microbiota (38). Specifically, foods with omega-3 polyunsaturated fatty acids (PUFA) and fiber can positively modify microbiota through the proliferation of bacteria with anti-inflammatory action. Both short-chain fatty acids (SCFA) obtained from the bacterial fermentation of dietary fibers, and PUFAs, contained in fish, have an anti-inflammatory function (39, 40). Nonetheless, we cannot exclude that disease-related symptoms such as mobility deficits and cognitive impairment in more disabled PwMS might have affected the access to healthier food, that usually require a more dedicated preparation rather than easy snacks or fast-foods, partly accounting for the observed relationship between food hubs intake and disability (41). However, in our sample of PwMS we have not recruited those with an EDSS higher than 5.5, also in order to restrain this potential bias.

### Relationship Between “Negative Foods” Lifestyle Factors and Disability

Our *post-hoc* analysis disclosed an independent predictive role for “negative foods,” exercise and smoking on motor disability. This finding suggests that not only the intake of “positive foods” is related to disability, but that the global composition of the dietary regimen as well as the lifestyle adopted potentially affect MS outcomes. Specifically, both smoking habit and low level of exercise were related to disability and fatigue independently from diet, as previously suggested (42, 43). Although reverse causality and simultaneity cannot be excluded when interpreting these associations, the relevance of a balanced dietary regimen, rich in “positive foods” and poor in “negative foods,” associated to a healthy lifestyle, might explain why studies exploring the beneficial effects of isolated dietary supplementations are often unsuccessful (44).

Our work is not without limitation. First, the cross-sectional design of our work limited the ability to explore the temporal relationship between lifestyle habits and changes in disability and fatigue over time, preventing us from drawing conclusions about direct causality. Second, we used a qualitative questionnaire to assess the adequacy to MeDi and food intake, intrinsically limiting the exact estimation of nutrients intake. We did not measure serum markers (i.e., antiflammatory or antioxidant) that could better reveal the association between nutrition intake and disability or fatigue if inserted in the network analysis, but on the basis of our results, future more comprehensive study on the matter might lead to further elucidation. Moreover, the use of family members as part of the control group might have limited the possibility to reveal real differences in dietary and lifestyle habits with PwMS. We have tried to minimize this occurrence introducing another set of controls in the HC group (healthcare staff). Indeed, the choice of family members as control group lie on the basis that family peers share characteristics such as socioeconomic and educational level with their relatives, that are known to influence diet behaviors (45).

## Conclusion

In conclusion, we identified a sufficient, although not optimal adherence to MeDi in our population. People living with MS showed a healthier dietary pattern than HC. Among food hubs, vegetables and fish were related to disability outcomes, which, in turn, were also predicted by intake of foods rich in saturated fats and alcohol, smoking and exercise. Our results confirm the association between diet, lifestyle and disability, suggesting that the modulation of these factors might affect MS outcomes. Further, our results suggest that, rather than the intake of a single food, the association of a food with the others is at the basis of the difference between PwMS and HC dietary habits. Future longitudinal studies applying integrated approaches should be planned to confirm these hypotheses, as the adoption of specific dietary regimens and exercise plans, could be used as complementary to the prescription of standard pharmacological therapies.

## Data Availability Statement

The raw data supporting the conclusions of this article will be made available by the authors, without undue reservation.

## Ethics Statement

The studies involving human participants were reviewed and approved by Ethical Committee board of Sapienza University of Rome at Sant'Andrea Hospital. The patients/participants provided their written informed consent to participate in this study.

## Author Contributions

FF, MP, CP, and SR: conceptualization. CP and SR: supervision and funding acquisition. FG, CP, and SR: project administration. All authors: methodology, formal analysis, investigation, data curation, writing, and have read and agreed to the published version of the manuscript.

## Funding

This study was partially supported by Sapienza University of Rome Italy Research Initiation Project n. RP120172B4168A93. This funding source had no role in the design of this study and did not have any role during its execution, analyses, interpretation of the data, or decision to submit results.

## Conflict of Interest

MP discloses travel/meeting expenses from Novartis, Roche and Merck, speaking honoraria from HEALTH&LIFE S.r.l. and honoraria for consulting services from Biogen and research grants from Baroni Foundation. CP has served on scientific advisory boards for Actelion, Biogen, Genzyme, Hoffmann-La Roche Ltd, Merck, Novartis, Sanofi, Teva, and has received consulting and/or speaking fees, research support and travel grants from Allergan, Almirall, Biogen, Genzyme, Hoffmann-La Roche Ltd, Merck, Novartis, Sanofi and Teva; SR has received honoraria from Biogen, Merck Serono, Novartis and Teva for consulting services, speaking and/or travel support. The remaining authors declare that the research was conducted in the absence of any commercial or financial relationships that could be construed as a potential conflict of interest.

## Publisher's Note

All claims expressed in this article are solely those of the authors and do not necessarily represent those of their affiliated organizations, or those of the publisher, the editors and the reviewers. Any product that may be evaluated in this article, or claim that may be made by its manufacturer, is not guaranteed or endorsed by the publisher.
